# Effect of extraction technique on chemical compositions and antioxidant activities of freeze-dried green pepper

**DOI:** 10.3389/fnut.2022.998840

**Published:** 2022-09-02

**Authors:** Chaohua Zhang, Fenglin Gu, Weicheng Hu, Guiping Wu, Weijun Chen, Conghui Dong, Zhiqiang Niu

**Affiliations:** ^1^College of Food Science and Engineering, Hainan University, Haikou, China; ^2^Sanya Research Institute of Chinese Academy of Tropical Agricultural Sciences, San Ya, China; ^3^Spice and Beverage Research Institute, Chinese Academy of Tropical Agricultural Sciences, Wanning, China; ^4^Hainan Key Laboratory for Biosafety Monitoring and Molecular Breeding in Off-Season Reproduction Regions, San Ya, China; ^5^Key Laboratory of Processing Suitability and Quality Control of the Special Tropical Crops of Hainan Province, Wanning, China; ^6^Jiangsu Collaborative Innovation Center of Regional Modern Agriculture & Environmental Protection, Huaiyin Normal University, Huaian, China

**Keywords:** freeze-dried pepper, pepper oleoresin, ultrasonic-microwave extraction, antioxidant activity, extraction kinetics

## Abstract

In this study, the yield, content of piperine, and antioxidant activity of pepper oleoresin obtained with the methods of maceration, ultrasound-assisted extraction (UAE), microwave-assisted extraction (MAE), and ultrasound-MAE (UMAE) were analyzed, and the microstructure of pepper residue was observed. For the yield and piperine content, the UMAE method had the best extraction capacity among the four methods. While, the oleoresin obtained with maceration had the highest total phenolic content, and the antioxidant activity of the oleoresin obtained by maceration was higher than that of the extracts acquired by UAE, MAE, and UMAE, and a high positive correlation was observed between the antioxidant activity and total phenolic content of the oleoresin obtained by these extraction methods. The ideal parameters for UMAE were an 80-mesh particle size and a 1 g/10 mL solid–liquid ratio. The kinetic parameters and models of the UMAE extraction process were also compared using first- and second-order models. The second-order kinetic equation with the lowest root mean square deviation and highest adjusted correlation coefficient proved to be more suitable for describing the extraction kinetics of pepper oleoresin. This study showed that UMAE is a fast, efficient, and cost-effective technique for the extraction of green pepper oleoresin.

## Introduction

Herbs and spices play an important role in human nutrition, and spices are natural food additives that are used to enliven and improve food flavor. Pepper (*Piper nigrum* L.), a common food additive and an important tropical spice crop in the world (1), is an essential spice in people's lives. Pepper is not only a food additive but also a medicine whose value has gained widespread attention in the medical field. In food production and processing, pepper is a common food flavoring with preservative and antioxidant effects, and it is widely used as a preservative spice for cured products, such as the storage of fresh pork (2).

Oleoresins are concentrated extracts derived from a variety of plant components, spices, and herbs. They are more complex extracts than essential oils because they include non-volatile components, colors, and irritant compounds, such as carotenoids and alkaloids, in addition to volatile chemicals (3). Given that they display a more complete aroma and flavor, oleoresins can be used in smaller quantities than essential oils and fragrances. The food industry is interested in oleoresin additions because they are rich in chemicals that offer aroma, flavor, color, and pungency. Spices offered as oleoresin and essential oils contribute to the product's value (4). Turmeric oleoresin is used for coloring, whereas pepper oleoresin is commonly employed for its spicy properties.

Pepper oleoresin, one of the main components of pepper, is an oily substance obtained by extracting pepper powder with organic solvents. Pepper oleoresin is a viscous dark green liquid with a spicy scent and flavor. It has a spicy and bitter flavor with a hint of lemon and clove. Its main components are volatile oil and piperine, containing the total aromatic components and pungent stimulating substances of peppers (5). Pepper oleoresin is utilized in a variety of foods, including meats, sauces, chutneys, soups, and snacks, due to its distinctive scent. The easy storage method and transportability of piper oleoresin enable its wide use in the food processing industry. Several studies have determined the functional health benefits of piperine, the main source of pungent substances in pepper oleoresin (6). It is reported that piperine exhibits a wide range of activities including anti-inflammation, anticancer, antimicrobial and anti-diabetic effect (7–10). It is also reported that piperine is a potent inhibitor of severe acute respiratory syndrome coronavirus 2 (COVID-19) though high biding affinity toward the RNA-binging site (11). Thus far, China has been mainly processing and producing the primary products of pepper, whereas most of the expensive deeply processed products, such as pepper oleoresin, pepper essential oil, and pepper capsules, rely on imports from abroad (12, 13).

Traditional extraction methods, such as Soxhlet and maceration, are simple to operate and offer the benefits of accuracy and precision in the extraction of piperine in samples; however, it presents several disadvantages such as higher extraction times and solvent consumption and bioactive compounds degradation (14). Ultrasound-assisted extraction (UAE), microwave-assisted extraction (MAE), and ultrasonic-MAE (UMAE) techniques have become well established. UMAE have the advantages and disadvantages of microwave and ultrasonic extraction methods to complement each other. Microwaves can effectively compensate for the lack of heat production by ultrasound, and ultrasound can effectively compensate for the uneven heating by microwave, thus making the UMAE more efficient compared with the other extraction methods (15). UMAE has a short extraction time, moderates the extraction temperature, avoids the use of excessive solvents, and has certain energy-saving advantages.

Previous literatures have compared the yields of various extraction methods for the extraction of oleoresin from pepper. According to their results, the new methods always have a higher piperine content or are less time-consuming than the conventional methods (16, 17). However, to the best of our knowledge, there is no available literature reporting the kinetics of pepper oleoresin extraction using ultrasound and microwave assisted extraction technologies.

This study aimed to compare innovative extraction methods with the conventional and investigate UMAE in depth to fit the kinetic model of UMAE extraction, optimize the extraction process of UMAE, and reduce the extraction cost. In the first part of this study, the extraction efficiency, antioxidant activity, and total phenolics of the pepper oleoresins was compared using maceration, UAE, MAE, and UMAE. In the second part, the effects of sample size and solid–liquid ratio on the yield of pepper oleoresin were investigated, and the extraction conditions were optimized. This study can provide an efficient alternative and “green” technique for the deep processing of pepper oleoresin.

## Materials and methods

### Materials and chemicals

Freeze-dried green pepper with a moisture content of 9.66 ± 0.12 g/100 g DW was obtained in July 2021 from Spice & Beverage Research Institute in Wanning (Hainan, China) and stored in an ultra-low temperature refrigerator (−80°C). Green pepper was powdered using a high-speed herbal crusher (Global Pharmaceutical Machinery Factory, Rui'an, China) and then passed through an 80-mesh sieve. The piperine and cyclohexanone standards used for identification and quantification were purchased from Aladdin Biochemical Technology (Shanghai, China). The analytical-grade ethanol used for extraction was purchased from Xilong Scientific Co., Ltd. (Guangdong, China). The high-performance liquid chromatography (HPLC)-grade organic solvents methanol and acetonitrile were purchased from Merck (Darmstadt, Germany). Other reagents were of analytical grade. The ultrapure water used in this study was purified by a Milli-Q system (Millipore, Milan, Italy).

### Extraction methods

#### Maceration

A total of 20 g powdered green pepper was placed in a 500 mL conical flask, and 200 mL anhydrous ethanol was added into the conical flask. The conical flask was sealed and placed in a water bath at the temperature of 60°C ranged from 120 to 720 min. The extract solvent was filtered and collected in brown reagent bottles sealed for further testing.

#### UAE

A total of 20 g green pepper powder was mixed with 200 mL anhydrous ethanol. The mixture was sonicated at room temperature (25 °C) by an ultrasonic microwave synergy extractor (CW-2000, Xintuo, Shanghai, China). The treatment time ranged from 15 to 90 min. The extract solvent was filtered and collected in brown reagent bottles sealed for testing.

#### MAE

A total of 20 g green pepper powder was mixed with 200 mL anhydrous ethanol. The mixture was treated by microwave using the CW-2000 extractor at a microwave temperature of 60°C and a microwave power of 300 W. The treatment time ranged from 3 to 18 min. The extract solvent was filtered and collected in brown reagent bottles sealed for testing. The MAE process consisted of two parts, including a 25 s microwave treatment and a 5 s interval.

#### UMAE

A total of 20 g green pepper powder was mixed with 200 mL anhydrous ethanol. The mixture was subjected to UMAE by the CW-2000 extractor with the ultrasonic function, a microwave temperature of 60°C, and a microwave power of 300 W. The treatment time ranged from 3 to 18 min. The extract solvent were filtered and collected in brown reagent bottles sealed for testing. The method was labeled as UMAE. For the kinetic analysis, different microwave powers ranging from 100 W to 500 W were used, and the extraction times ranged from 3 min to 18 min. Different parameters were also set for the solid–liquid ratio and sample powder size.

#### Yield

The extracts were filtered into a constant-weight rotary evaporation flask and concentrated using a rotary evaporator (R-215, Buchi, Switzerland) in a constant-temperature water bath at 55°C until no solution dripped out. The flask was then dried to a constant weight in a vacuum dryer at 50°C. The contents of the flask were then converted into pepper oleoresin.

Four methods were used for the extraction of green pepper powder, and the yield of pepper oleoresin was calculated as follows:


(1) $$\begin{array}{l} C\left(\%\right)=\frac{y_{a}\times 100}{y_{b}} \end{array}$$

where *y*_*a*_(g) and *y*_*b*_(g) are the weight of pepper oleoresin and the DW basis of freeze-dried green pepper, respectively, and *C*(%) is the yield of pepper oleoresin.

#### Piperine content

A HPLC (Agilent 1260 series HPLC, USA) equipped with a ZORBAX SB-C18 column (4.6 × 100 mm, 3.5 μm, Agilent) was used to determine the piperine content, as described by Lee's work (18) with slight modification. First, 20 mL anhydrous ethanol was poured into a rotary evaporator flask to dissolve oleoresin, and 1 mL mixture was drawn into a 100 mL brown volumetric flask and diluted to the scale with anhydrous ethanol. The dilutions were filtered through a 0.45 μm filter membrane and quantified by the external standard method. The following mobile phases were used: A was methanol, B was acetonitrile, and C was water. The ratio was 80% A, 10% B, and 10% C (V/V/V) with a flow rate of 0.5 mL min^−1^, column temperature of 30 °C, detector wavelength of 343 nm, and injection volume of 10 μL.

The standard solutions of piperine at different mass concentrations (0.4–2.0 mg/L) were prepared with ethanol and filtered through a 0.45 μm filter membrane. Next, 10 μL standard solutions were injected into the HPLC equipment, and the standard curves were determined and plotted. Data analysis was performed using an Agilent chromatography management system (OpenLab CDS C.01.07, Agilent Technologies). All processes were protected from light and repeated thrice.

### Analysis of total phenols

Phenolic content was determined by the Folin–Ciocalteu method (19). The dried concentrated oleoresin in the round bottom flask was redissolved, removed to volumetric flask, and fixed with ethanol to 20 ml, and 100 μl oleoresin solution was obtained and diluted to 10 ml with ethanol. A total of 1 mL dilution was mixed with 1 mL Folin-Ciocalteu reagent and 3 mL 7.5% Na_2_CO_3_ solution. After 60 min incubation at room temperature, a 200 μL aliquot was added into individual well of a 96-well plate and measured at 765 nm using a Hybrid Multi-Mode Microplate Reader (BioTek, SynergyH1, USA). The mass fraction of total phenols in the sample was expressed as gallic acid equivalent (mg GAE/g DW).

### Scanning electron microscopy analysis

The microstructural changes in the pepper residue before and after extraction by maceration, ultrasound, microwave, and ultrasonic-microwave leaching were analyzed by SEM (Phenom, Phenom Prox, Netherlands). Briefly, a small amount of the sample was placed on a carbon tape and attached to the sample tray. Then, a thin layer of gold was sputtered. The structure was examined using a scanning electron microscope at various magnifications under standard vacuum conditions with an accelerating voltage of 10.0 kV and a secondary electron detector (20).

### Antioxidant activity

#### DPPH radical-scavenging assay

The DPPH radical-scavenging capability was referenced from reported literature with slight modifications (21). The dried concentrated oleoresin in the round bottom flask was redissolved, removed to volumetric flask, and fixed to 20 ml with ethanol. A total of 150 μL sample solution and 150 μL DPPH working solution for the sample group, 150 μL sample solution and 150 μL 80% methanol for the control group, and 150 μL 80% methanol and 150 μL DPPH working solution for the blank group were prepared. The reaction was carried out at room temperature (25 °C) for 30 min, and the samples were centrifuged for 5 min at room temperature. A 200 μL aliquot of each solution was added to individual wells of a 96-well plate, and the absorbance values were read at 517 nm and labeled as *A*_*control*_, *A*_*sample*_, and *A*_*blank*_. The amount of antioxidant Trolox obtained from the standard curve (μg Trolox/mL) was used to express the DPPH radical-scavenging capability of the samples by plotting the standard curves for different concentrations of Trolox. The scavenging of DPPH radicals is calculated by the following formula:


(2) $$\begin{array}{l} DPPH\;scavenging\;capacity(\%)=\left[1-\frac{\left(A_{sample}-A_{control}\right)}{A_{blank}}\right]\\ \;\times 100 \end{array}$$

where *A*_*control*_, *A*_*sample*_, and *A*_*blank*_ are the absorbances of the control, sample, and blank, respectively. The experiment was repeated thrice.

#### ABTS radical-scavenging assay

The ABTS free radical-scavenging capacity test was referenced from Chanioti's method (22). The dried concentrated oleoresin in the round bottom flask was redissolved, removed to volumetric flask, and fixed to 20 mL with ethanol. The ABTS radical-scavenging capability was determined by mixing 10 μL sample solution and 190 μL ABTS working solution. The mixture was left to stand for 6 min at room temperature, and the absorbance value was read at 734 nm and labeled as *A*_*sample*_. The sample solution with 190 μL anhydrous ethanol was used as the control group, and 10 μL anhydrous ethanol 190 μL working solution of ABTS was used as the blank group. The reaction was carried out at room temperature for 6 min, and the absorbance values were read at 734 nm and labeled as *A*_*control*_ and *A*_*blank*_, respectively. The ABTS radical-scavenging capability of the samples was expressed in terms of the amount of antioxidant Trolox (μg Trolox/mL) obtained from the standard curve after plotting the standard curves for different concentrations of Trolox. The scavenging of ABTS radical was calculated using the following equation:


(3) $$\begin{array}{l} ABTS\;scavenging\;capacity\;(\%)=\left[1-\frac{\left(A_{sample}-A_{control}\right)}{A_{blank}}\right]\\ \;\times 100 \end{array}$$

where *A*_*control*_, *A*_*sample*_, and *A*_*blank*_ are the absorbances of the control, sample, and blank, respectively. The experiment was repeated thrice.

### Overall score of the four extraction methods

The yields of pepper oleoresin, piperine content, and total phenolic content were utilized as indicators, and the data for each indication was standardized and weighted before being analyzed. The weights were evaluated using reduced factor analysis in Statistical Package for the Social Sciences (SPSS) (version 26.0, IBM, New York, USA), and the weighted scores of the samples were produced as an evaluation index of sample quality. The higher the overall score, the better the quality. The formula for calculating the overall score is as follows:


(4) $$\begin{array}{l} Overall\;Score=F_{oleoresin}^{*}a+F_{piperine}^{*}b+F_{phenol}^{*}c \end{array}$$

where *F*_*oleoresin*_, *F*_*piperine*_, and *F*_*phenol*_ denote of oleoresin yield, piperine content, and total phenolic content of each sample, respectively, and scores a, b, and c represent their weights.

### Kinetic model

#### First-order extraction kinetic model

The differential form of the first-order kinetic model (23) can be written as Eq. (5):


(5) $$\begin{array}{l} \frac{dy_{t}}{d_{t}}=k_{1}\left(y_{s}-y_{t}\right) \end{array}$$

where *k*_1_ (L/g·min) is the extraction rate constant, *y*_*s*_ (%) represents the yield of pepper oleoresin at saturation, and *y*_*t*_ (%) denotes the yield of pepper oleoresin at a given extraction time (t, min). Eq. (6) is integrated under the boundary conditions of *y*_*t*_ = 0 at *t* = 0 and *y*_*t*_ = *y*_*t*_ at *t* = *t*:


(6) $$\begin{array}{l} ln\left(\frac{y_{S}}{y_{s}-y_{t}}\right)=k_{1}t \end{array}$$

Eq. (6) may be rearranged to obtain a linear form:


(7) $$\begin{array}{l} log\left(y_{s}-y_{t}\right)=log\left(y_{s}\right)-\frac{k_{1}}{2,303} \end{array}$$

Plotting *log*(*y*_*s*_ − *y*_*t*_) vs. t for different experimental conditions, *k*_1_ can be obtained from the slope and the concentration at saturation (*y*_*s*_) from the intercept.

#### Second-order extraction kinetic model

The second-order kinetic equation (20) for the extraction rate can be written as follows:


(8) $$\begin{array}{l} \frac{dy_{t}}{d_{t}}=k_{2}{\left(y_{s}-y_{t}\right)}^{2} \end{array}$$

where *k*_2_ (L/g·min) is the second-order extraction rate constant, *y*_*t*_ (%) represents the yield of pepper oleoresin at a given extraction time (t, min), and *y*_*s*_ (%) refers to the yield of oleoresin at saturation.

By integrating Eq. (8), the following formula was obtained:


(9) $$\begin{array}{l} \frac{dy_{t}}{{\left(y_{s}-y_{t}\right)}^{2}}=k_{2}dt \end{array}$$

At the boundary condition of *t* = 0 *y*_*t*_ = 0 and at *t* = *t y*_*t*_ = *y*_*t*_ integrating Eq. (9), the following integrated rate equation was obtained:


(10) $$\begin{array}{l} y_{t}=\frac{y_{s}^{2}k_{2}t}{1+y_{s}k_{2}t} \end{array}$$

By transforming Eq. (11), a linear form shown in Eq. (11) can be obtained, and the extraction rate can be written as Eq. (12):


(11) $$\begin{array}{l} \frac{t}{y_{t}}=\frac{1}{y_{s}^{2}k_{2}}+\frac{t}{y_{S}} \end{array}$$

(12) $$\begin{array}{l} \frac{y_{t}}{t}=\frac{1}{\left(1/k_{2}y_{s}^{2}\right)+\left(t/y_{s}\right)} \end{array}$$

The initial extraction rate, *h*, which is $\frac{y_{t}}{t}$ when *t* approaches 0, can be defined as follows:


(13) $$\begin{array}{l} h=ky_{s}^{2} \end{array}$$

The oleoresin extraction rate at any time can be presented as Eq. (13):


(14) $$\begin{array}{l} y_{t}=\frac{t}{\left(1/h\right)+\left(t/y_{s}\right)} \end{array}$$

The initial extraction rate *h*, extraction capacity *y*_*s*_, and the second-order extraction rate constant *k*_2_ can be determined experimentally from the slope and intercept by plotting *t*/*y*_*t*_ vs. *t*.

The root mean square deviation (RMSD) and the adjusted correlation coefficient ($R_{adj}^{2}$) were used to evaluate the models. Low RMSD and high $R_{adj}^{2}$ values indicate a good fit.


(15) $$R^{2}=1-\frac{(n-1)\;\times \sum_{i=1}^{n}{\left(y_{pv}-y_{ev}\right)}^{2}}{(n-1-m)\;\times \sum_{i=1}^{n}{\left({\bar{y}}_{ev}-y_{ev}\right)}^{2}}$$

(16) $$\begin{array}{l} RMSD=\sqrt{\frac{1}{n}\overset{n}{\underset{i=1}{\sum }}{\left(y_{pv}-y_{ev}\right)}^{2}} \end{array}$$

where *y*_*pv*_ and *y*_*ev*_ are respectively the predicted and experimental yields of pepper oleoresin, the number of independent variables is *m*, and the number of experimental data is *n*.

### Statistical analysis

All analyses were carried out in triplicate, and the results were expressed as means ± standard deviation (SD). Analysis of variance by SPSS version 26.0 (IBM, New York, USA) was used to test the model significance and calculate the optimal conditions for pepper oleoresin extraction. The extraction kinetics curves of each method based on the first-order and second-order kinetic models were fitted using OriginPro 2021 software (OriginLab, Massachusetts, USA). Statistical significance was set at *p* < 0.05.

## Results and discussion

### Comparison of the four kinds of extraction methods

#### Pepper oleoresin yield

**Figure 1:**
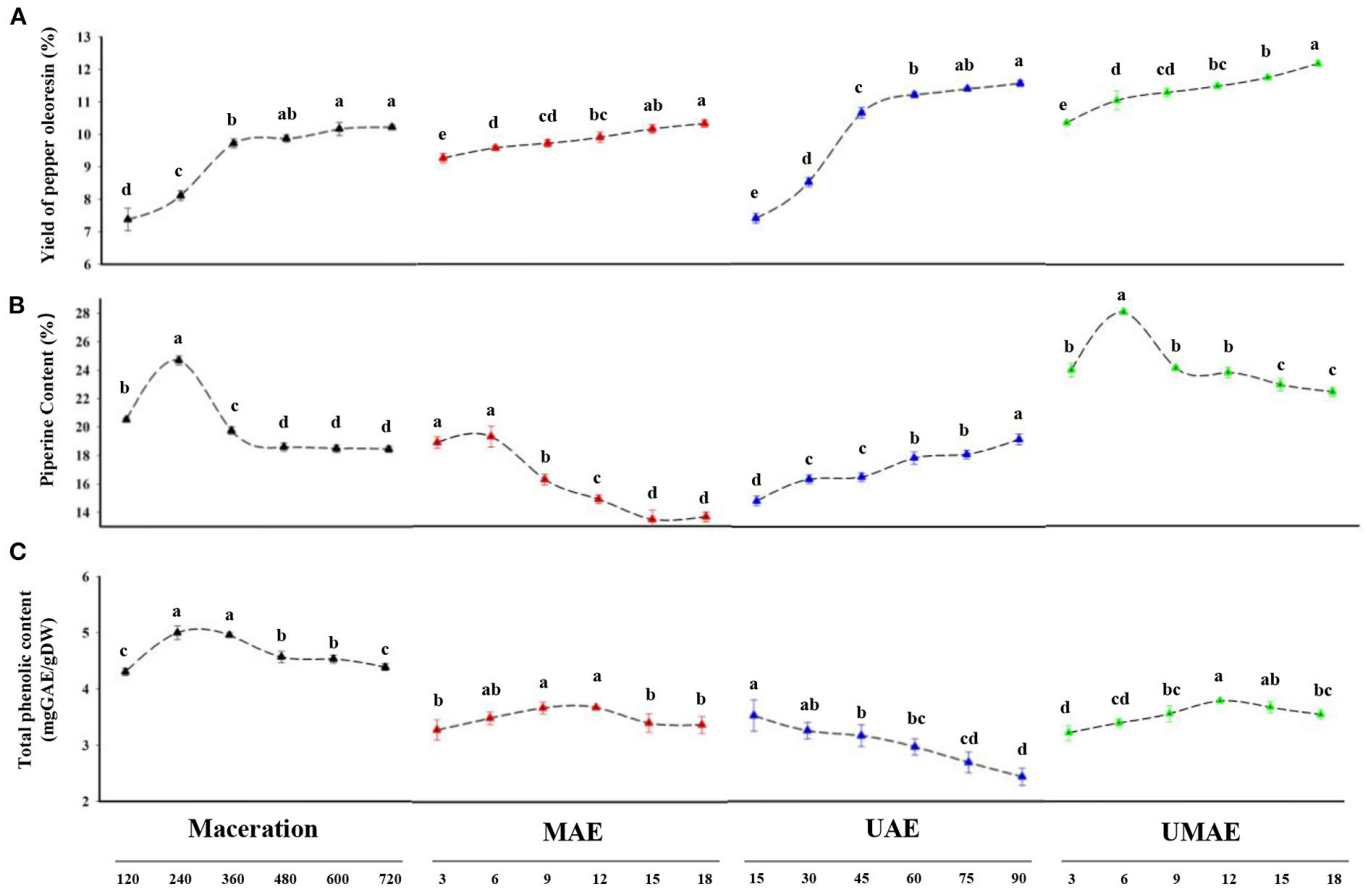
Comparison of yield, piperine content, and total phenolic content of extract from maceration, UAE, MAE, and UMAE at different extraction times (3–720 min). **(A)** Yield of pepper oleoresin. **(B)** Piperine content of pepper oleoresin. **(C)** Total phenolic content expressed mg GAE/g DW. Different letters in the same treatment indicate a statistical difference (*p* < 0.05).

Figure 1A shows the yields of piper oleoresin by the four extraction methods based on the extraction time. UMAE had the highest extraction yield of 12.14% and shortest extraction time (18 min) compared with the other three methods because the process of extraction reached equilibrium. UAE showed a higher yield (11.40%), the MAE yield exhibited no significant difference from maceration (10.22%), whereas the extraction time required to reach equilibrium (90 min for UAE and 18 min for MAE) was considerably less than that required for maceration (600 min for maceration). UAE and MAE were favorable for the extraction of piper oleoresin.

Given that most of the active ingredients in plants are intracellular products, cell fragmentation is required for extraction. In addition to free diffusion, microwave radiation and ultrasonic cavitation will increase the degree of cell fragmentation, thus affecting the extraction process (24). With regard to maceration, ethanol can cause cell breakage or deformation by penetrating the cell and causing dehydration or dissolving cell membranes. Ultrasound uses cavitation to improve the permeability of the cell membrane and cell wall, facilitating the dissolution of chemical components in the plant in the solvent (25). The microwave radiation effect improves the flow rate and mass transfer rate of the solvent with the increase in temperature, and the volatility and diffusion coefficient of active substances increase, which improves the extraction rate of active plant components (26). Similar to the present study, ultrasound-microwave technique had better yield for lavender essential oil (27), pectin from jackfruit peel (28) and bioactive compounds from brown macroalgae (29).

#### Piperine content

Figure 1B shows the results of piperine content in the extracts of pepper powder obtained by different extraction methods. The content of piperine in the oleoresins of extracts acquired by maceration, MAE, and UMAE increased first and then decreased with the extension of extraction time, whereas the content of piperine in the oleoresin obtained by UAE increased to its maximum value (19.25 %) at 90 min and then decreased with the extension of extraction time. The piperine content in oleoresin was higher (25.66%) during 240 min of maceration, and the piperine content in the oleoresin extracted by MAE (19.81%) was significantly lower than that obtained with UMAE. The highest piperine content (28.32%) was obtained by UMAE extraction. This result can be due to the microwave's unique thermal effect, which caused the temperature to rise, affecting the difference in piperine content with extraction time amongst the four methods. As the temperature rose, internal membrane components may be withdrawn more rapidly and easily, speeding up the breakout of piperine in oleoresin. However, more volatile components were reduced or eliminated with the rise in temperature (30). Piperine is a relatively unstable substance that is sensitive to light, heat, and oxygen. Temperature can improve the release of chemicals bound to cell wall macromolecules by increasing the solubility of the component (31). Thus, the extraction temperature is crucial for the extraction of oleoresin and piperine.

The yield of pepper oleoresin was favorably linked with the extraction temperature; however, the piperine content was negatively connected with the extraction temperature, indicating that piperine is affected by the extraction temperature (32). The UAE was only ultrasonically cavitated in the trials, and it was held at a low extraction temperature to protect piperine from thermal degradation but not from the extraction of volatile compounds in pepper, such as the pepper oleoresin examined in this article. A similar study also found that combination of microwave- and ultrasound-assisted extraction can improve the yield of oligosaccharides sweet potatoes (33).

#### Total phenolic content

The antioxidant effects of plant phenolics have been proved for the prevention of coronary heart disease, cancer, and age-related degenerative brain diseases (34). Furthermore, phenolic substances have been linked to antioxidant activity and have been shown to have a role in lipid antioxidation. Figure 1C shows that the total phenolic content of oleoresin extracted by maceration was the highest and consistently higher than those of the other three methods. The total phenolic contents of oleoresin extracted by MAE and UMAE increased and then decreased as the extraction time increased. In addition, the total phenolic contents of the extracts treated with MAE and reached the maximum at the extraction times 9 (3.71 mg GAE/g DW) and 15 (3.86 mg GAE/g DW) min, respectively. However, the total phenolic content of oleoresin extracted by UAE decreased as the extraction time increased. Possibly, the ultrasonic waves destroyed the structure of phenolics; similar conclusions were obtained in a study on the changes in gallic acid mediated by ultrasound in a model extraction solution by Zhang et al. (35). Ultrasonic treatment induced the significant degradation of phenols dissolved in the ethanol solution, resulting in the decreased total phenolic content of the oleoresins extracted by UAE. At 15 min extraction time, the total phenolic content of UAE (3.75 mg GAE/g DW) reached its maximum. With the extension of the extraction process, the total phenolic content of UAE decreased. When maceration was utilized at 240 min, the highest total phenol content was obtained (4.99 mg GAE/g DW), which was the highest value obtained for the four extraction procedures. It can be explained that microwave and ultrasound extraction way might have sped up the fragmentation of the pomace tissues and made it easier for the phenolics to drain from the cells (36).

#### Microstructure of pepper powder

**Figure 2:**
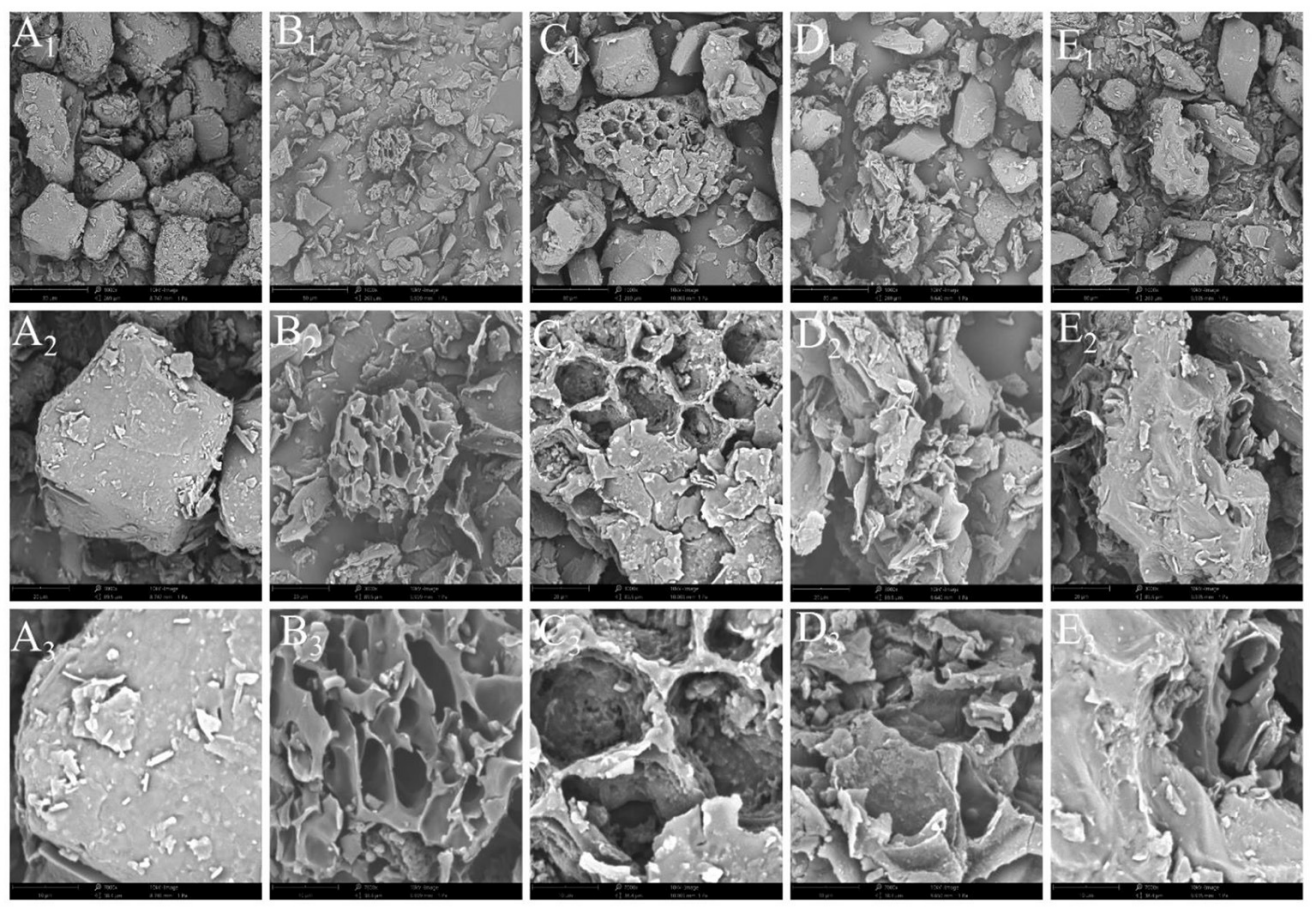
Microstructure of pepper powder **(A)** and maceration **(B)**, UAE **(C)**, MAE **(D)**, and UMAE **(E)** after treatment. Subscripts 1, 2, and 3 represent magnifications of 1000x, 3000x, and 7000x, respectively.

The surface morphology of pepper powder before and after extraction was examined using SEM to ascertain the mechanisms of maceration extraction, UAE, MAE, and UMAE during pepper oleoresin extraction. Although several cells were dehydrated or killed during the lyophilization procedure, the cell structure of a substantial number of tissue cells (before extraction) was visible (Figures 2A_1−3_) (27). The tissue cells of the samples handled with different extraction procedures, on the other hand, were destroyed to varying degrees (Figures 2B_1−3_). Evident holes were observed in the pellets following ultrasonication (Figures 2C_1−3_), more severe breaking occurred in the microwave-treated pellets (Figures 2D_1−3_), and essentially no intact cells in the pellets were obtained after synergistic ultrasonic microwave treatment (Figures 2E_1−3_) (37). The ultrasonic-microwave co-treatment caused extensive cell breakage and bioactive chemical release into the solvent (38).

### Antioxidant activity

#### DPPH free-radical scavenging assay

**Figure 3:**
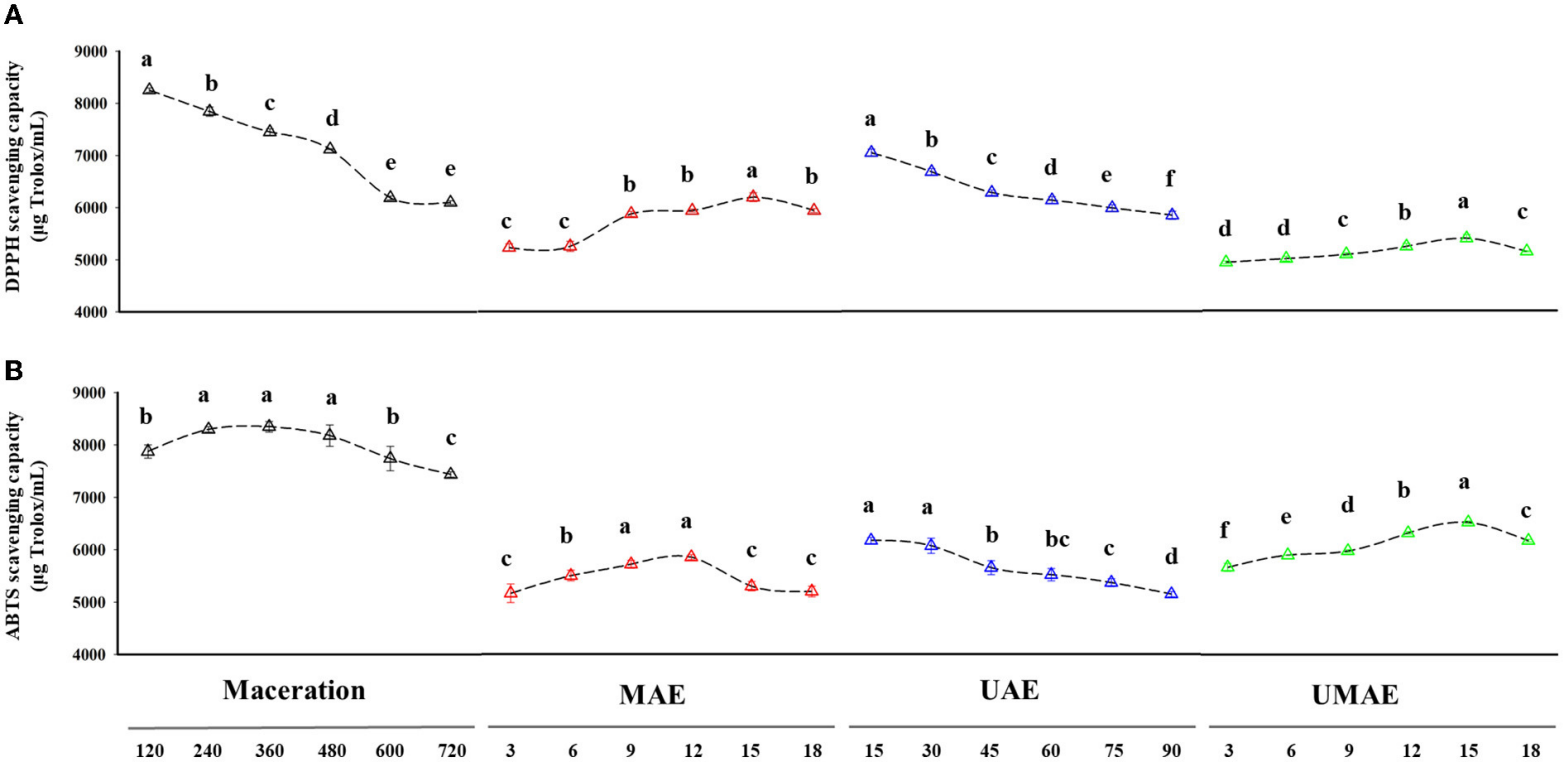
Antioxidant capacity of oleoresins obtained by different extraction methods. **(A)** DPPH radical-scavenging capacity. **(B)** ABTS radical-scavenging capacity. Different letters in the same treatment indicate a statistical difference (*p* < 0.05).

Several *in vitro* assays were utilized to assess the antioxidant activity of pepper oleoresin extracted using different procedures, and the amount of the antioxidant Trolox was used to calculate the samples' DPPH and ABTS free radical-scavenging capacities (Figures 3A,B).

DPPH scavenging capacity analysis is a simple chemical analysis of the reaction of antioxidants by scavenging DPPH radicals, which always results in a decrease in absorbance at 517 nm (39). The extract obtained by maceration at 240 min (Figure 3A) had the highest DPPH-scavenging capability (8,291.43 μg Trolox/mL), and it notably exceeded those of the other three extracts. The DPPH-scavenging capability of maceration extract declined with the increase in extraction time, although it was still higher than the highest value of UMAE (5,463.87 μg Trolox/mL). The DPPH-scavenging capacity of the extract of UAE decreased with the increase in extraction time and was the best at 15 min (7,108.46 μg Trolox/mL). The DPPH-scavenging capacity of the extracts of MAE and UMAE increased and then decreased with the increase in extraction time. At 15 min, the extract of MAE had the best DPPH radical-scavenging capacity (6,239.55 μg Trolox/mL). Similarly, the extract of UMAE had the best DPPH-radical scavenging capacity at 15 min (5,463.867 μg Trolox/mL). Previous literature reported that ultra-micro extraction techniques can also produce high yield of edible oils with high antioxidant capacity from the seeds of *Allanblackia parviflora* (37). The results of DPPH radical-scavenging capacity and total phenolic content of the extracts obtained from the four extraction methods treatments were highly correlated. The decrease in antioxidant capacity can be attributed to the high temperature and prolonged ultrasonic treatment, which led to the destruction and decomposition of the structure of several active substances.

#### ABTS assay

ABTS staining assay is another extensively used technique for the evaluation of the antioxidant activity of different substrates *in vitro*. As shown in Figure 3B, the extract of maceration showed the best ABTS radical-scavenging capacity, which reached the maximum (8,488.59 μg Trolox/mL) in the extract at 120 min and then decreased with the extended extraction time probably due to the destruction of several active substances as a result of prolonged heating. The ABTS radical-scavenging capacity of the extract of UAE decreased with the increase in extraction time and reached a maximum (6,216.69 μg Trolox/mL) at the beginning of extraction (15 min). The ultrasound treatment possibly disrupted the structure of the active substance, which resulted in a decrease in ABTS scavenging capacity. The ABTS radical scavenging capacity of the extracts of MAE and UMAE increased and then decreased with extraction time. The extract of UMAE reached the maximum ABTS radical-scavenging capacity (6,571.77 μg Trolox/mL) at 15 min. The scavenging capacity of UMAE was better than that of MAE and UAE due to the coupling effect of ultrasound and microwave on the sample increasing the ABTS scavenging capacity of the sample with time (40).

#### Overall score

The effects of the four extraction methods on the yield of oleoresin, piperine content, and total phenol content were compared and showed inconsistent results. Thus, oleoresin yield, piperine content, and total phenol content were obtained for SPSS factor analysis and analyzed for a preliminary judgment on the advantages and disadvantages of the four extraction methods. The overall scores were determined using the weights coefficient of the three factors, which were 46.64 % (oleoresin yield), 40.75% (piperine content), and 12.61% (total phenol content) (41). Table 1 shows that UMAE had the greatest overall score, indicating that the oleoresin extracted by UMAE was of the highest quality and that UMAE was the best extraction procedure.

**Table 1:** Comparison of factors and the overall score of oleoresins extracted by four kinds of methods.

**Indicator**	**Weight (%)**	**Maceration**	**UAE**	**MAE**	**UMAE**
*F*_oleoresin_	46.64	49.19 ± 3.55^c^	87.18 ± 0.66^a^	45.35 ± 1.26^d^	81.53 ± 0.70^b^
*F*_piperine_	40.75	43.21 ± 1.521^b^	39.07 ± 1.16^c^	38.47 ± 0.93^c^	99.26 ± 0.94^a^
*F*_phenol_	12.61	12.67 ± 1.07^b^	0.76 ± 0.86^c^	39.96 ± 1.41^a^	39.61 ± 0.91^a^
Overall score	100	42.15 ± 1.19^c^	56.68 ± 0.13^b^	41.86 ± 0.51^c^	83.47 ± 0.58^a^

Analytical results are the means ± standard deviation (*n* = 3). Different letters in a row indicate significant differences in the mean (*p* < 0.05). UAE-ultrasonic assisted extraction; MAE-microwave assisted extraction; UMAE–ultrasonic-microwave-assisted extraction.

### Effect of particle size and solid–liquid ratio on pepper oleoresin extraction

The effect of particle size and solid–liquid ratio on the yield of piper oleoresin was not examined in the first part. The optimal particle size and solid–liquid ratio should be determined for the better characterization of extraction kinetics.

**Figure 4:**
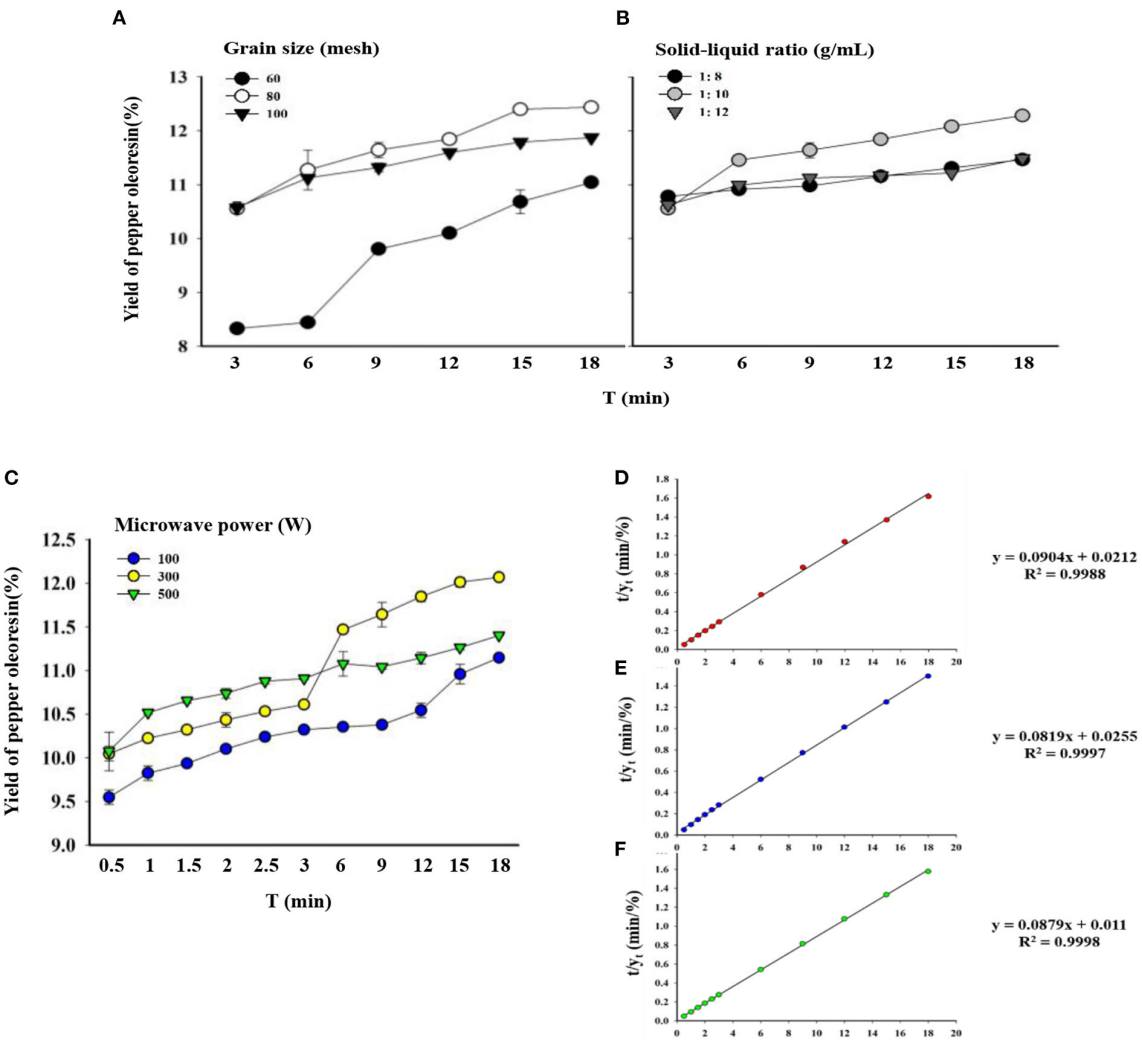
Yields of pepper oleoresin under different extraction conditions and second-order extraction kinetics for different microwave powers. **(A)** Different particle sizes. **(B)** Different solid–liquid ratios. **(C)** Different microwave powers (solid–liquid ratio fixed at 1 g/10 mL and particle size fixed at 80 mesh). **(D)** Second-order extraction kinetics of 100 W. **(E)** Second-order extraction kinetics at 300 W. **(F)** Second-order extraction kinetics at 500 W.

In this study, extraction was performed by using three different particle sizes (60, 80, and 100 mesh) of pepper powder and three different solid–liquid ratios (1/8, 1/10, and 1/12 g/mL) at z microwave power of 300 W and anhydrous ethanol (42); the results are shown in Figures 4A,B.

When the particle size of the pepper powder was 60 mesh, the greatest oleoresin yield was 11.13%. The low extraction yield may be due to the insufficient crushing of pepper, which is not conducive to the extraction of useful substances. The yield of oleoresin increased with the decrease in the mesh size. The oleoresin yield reached 12.51%, which was the maximum yield when the pepper particles reached 80 mesh. This finding may be the reason why at the smaller the particle size of the raw material, the more fully obtained extraction solvent can contact the raw material's surface area, the higher the degree of destruction of the plant cell wall, the greater the solvent penetration capability, and the easier the extraction of effective substances, resulting in a higher oleoresin yield (43, 44). When the particle size was 100 mesh, the greatest extraction rate was 11.9%, which can be related to the extremely fine raw materials agglomerating and hindering the flow of beneficial chemicals and increasing the difficulty of crushing.

The solid–liquid ratio has a major effect on piper oleoresin yield. When the solid–liquid ratio was changed from 1/8 g/ml to 1/10 g/ml, the extraction yield increased, but as the solid–liquid ratio decreased further, the yield declined. According to previous studies, the yield of coffee oil increased as the solid-to-liquid ratio decreased until it reached the maximum extraction rate (20). When the solid–liquid ratio was decreased beyond 1/12 g/ml, the yield was lower due to the diminished ultrasonic energy dispersion in the solvent, which hampered the dissolution of active component outflow. The same trend was observed for microwave extraction of medicinal oleoresin from black and white pepper (45) and with use of the UAE method (46).

### Kinetics of pepper oleoresin extraction

In this study, an appropriate model should be used to analyze the UMAE process. To select the best model for describing the UMAE of green pepper oleoresin, the extraction process was performed at three particle sizes (60, 80, and 100 mesh) and three solid-to-liquid ratios (1/8, 1/10, and 1/12 g/mL). First-order and second-order kinetic models were used to fit the experimental data. Table 2 presents the extraction capacity y_s_, $R_{adj}^{2}$, and RMSD. The second-order kinetic equations had higher $R_{adj}^{2}$ values (0.9937–0.9998) than the first-order kinetic equation. Furthermore, the second-order kinetic equation's RMSD values (0.0066–0.0337) were notably lower than those of the first-order kinetic equation. Thus, the second-order kinetic equation is the best mathematical equation to represent the kinetics of UMAE of green pepper oleoresin (47).

**Table 2:** RMSD and $R_{adj}^{2}$ values for first-order kinetics and second-order kinetics under the extraction conditions studied.

**Factors**		**First-order model**			**Second-order model**		
		**y_S_ (%)**	**RMSD**	** $R_{adj}^{2}$ **	**y_S_ (%)**	**RMSD**	** $R_{adj}^{2}$ **
Particle size (mesh)	60	10.361	0.1112	0.9128	12.069	0.0337	0.9937
	80	11.996	0.0184	0.9974	12.980	0.0131	0.9989
	100	11.576	0.0232	0.9909	12.209	0.0066	0.9998
Solid-liquid ratio (g/mL)	1:8	11.153	0.0126	0.9917	11.614	0.0097	0.9995
	1:10	11.918	0.0725	0.9748	12.657	0.0080	0.9996
	1:12	11.210	0.0287	0.9956	11.589	0.0098	0.9995

The optimal conditions for the rapid extraction of freeze-dried green pepper oleoresin by UMAE were determined by single-factor optimization experiments. The extraction process was carried out under ideal circumstances at three different microwave powers (100, 300, and 500 W) for the kinetic study. The extraction of pepper oleoresin showed an evident second-order process the extraction rate of oleoresin increased and then stabilized with the increase in extraction time (48). As shown in Figure 4C, first, at the beginning of the extraction process, increasing the microwave power allowed the solid–liquid system to absorb more energy and the temperature to rise rapidly, which can accelerate the solvent–solid matrix interaction. The molecular mobility and diffusion coefficients increased dramatically as the temperature rose.

Furthermore, when the microwave power increases, the boiling point of the combination is achieved faster, and the boiling intensity increases. Given that the boiling solvent causes friction between the substrate and the solvent, the solute is washed away from the surface and fractures the plant substrate. At this point, the majority of the extraction occurs. Second, a slower phase, during which internal diffusion is slower than exterior diffusion, serves as the limiting process. The microwave energy speeds up molecular movement and internal diffusion, causing a breach in the plant material's structure. Given that the substrate has been damaged, it becomes softer, allowing more solvents to infiltrate the plant matrix (49).

The experimental results and the second-order kinetic model were consistent, indicating that the ethanol extraction of green pepper oleoresin followed the previously described model (20). The analytical results of the experiment were plotted t/y_t_ vs. time using a second-order model (Figures 4D–F). Therefore, the saturation extraction capacity y_s_, the extraction rate constant k, the initial extraction rate h, and the adjusted correlation coefficient $R_{adj}^{2}$ were determined at different microwave powers according to the linear fitting equation given by Origin software (OriginLab, Massachusetts, USA) (y = ax+b, where y = t/y_t_, a = 1/y_s_, b = ${1/\text{ky}}_{\text{s}}^{2}$, and x is the extraction time).

Table 3 shows the parameters of the second-order kinetics. In regard to the second-order extraction rate constant (k) obtained from the second-order kinetic model, the second-order extraction rate constant (k) for microwave power of 500 W was notably higher (2.66 times) than that of 300 W. The initial extraction rate (h) for microwave power of 500 W was considerably higher (2.31 times) than that of 300 W. In addition, microwave power of 300 W extraction capacity value (y_s_) was slightly higher than 500 W (1.07 times). Although the y_s_ of oleoresin extracted by 500 W was slightly lower than that of 300 W, the k and h values of 500 W were evidently higher than that of 300 W. The results showed that the optimal microwave power for the extraction of green pepper oleoresin by UMAE was 500 W, and all the predicted values were consistent with the experimental finding with a certain accuracy. The model can be used to predict the extraction process of green pepper oleoresin, thus optimizing the extraction process and reducing the extraction cost.

**Table 3:** Second-order kinetic model parameters for UMAE of pepper oleoresin at different microwave powers.

**Microwave power**	**Slope**	**y_S_ (%)**	**Intercept**	**h**	**k**	$R_{adj}^{2}$
100W	0.0904	11.063	0.0213	47.0588	0.3845	0.9988
300W	0.0819	12.209	0.0255	39.2311	0.2632	0.9997
500W	0.0879	11.373	0.0111	90.4977	0.6997	0.9998

## Conclusion

In this study, the differences in pepper oleoresin extracted by the four extraction methods were compared by experimental analysis, and a kinetic model was fitted to the UMAE method using two models. When the yields of pepper oleoresin and piperine contents were compared, the UMAE method had the best extraction capacity of the four methods. UAE and MAE yielded slightly higher yields of oleoresin than maceration, and both methods consumed considerably less time to extract than the maceration method. The oleoresin obtained with maceration had the highest total phenolic content of 4.99 ± 0.12 mg GAE/g DW, and that obtained with UAE had the lowest total phenolic content of 2.42 ± 0.15 mg GAE/g DW. However, the total phenolic contents of the oleoresins obtained by MAE and UMAE were close. In the DPPH and ABTS radical-scavenging capacity assays, the antioxidant activity of pepper oleoresin by maceration was higher than that of the extracts of UAE, MAE, and UMAE, and a high positive correlation was observed between the antioxidant activity and the total phenolic content of the oleoresin obtained by the four extraction methods. The overall scores through the weights coefficient of the main factors verified that UMAE had the greatest overall score, indicating that the oleoresin extracted by UMAE was of the highest quality and that UMAE was the best extraction procedure. These findings demonstrated the potential of UMAE as a considered promising technique for the extraction of spice oleoresin.

## Data availability statement

The raw data supporting the conclusions of this article will be made available by the authors, without undue reservation.

## Author contributions

FG and WH contributed to design of the study. CZ responsible for document retrieval and manuscript writing. WH and WC responsible for making illustrations and tables. FG, WH, CZ, CD, GW, and ZN contributed to manuscript revision. All authors read and approved the submitted version.

## Funding

This work was supported by the National Key R&D Program of China (2020YFD1001200) and Central Public-interest Scientific Institution Basal Research Fund for Chinese Academy of Tropical Agricultural Sciences (No. 1630142022011).

## Conflict of interest

The authors declare that the research was conducted in the absence of any commercial or financial relationships that could be construed as a potential conflict of interest.

## Publisher's note

All claims expressed in this article are solely those of the authors and do not necessarily represent those of their affiliated organizations, or those of the publisher, the editors and the reviewers. Any product that may be evaluated in this article, or claim that may be made by its manufacturer, is not guaranteed or endorsed by the publisher.

## References

[1] Meghwal M Goswami TK. Piper nigrum and piperine: an update. Phytother Res. (2013) 27:1121–30. 10.1002/ptr.4972 23625885

[2] Zhang J Wang Y Pan D-D Cao J-X Shao X-F Chen Y-J. Effect of black pepper essential oil on the quality of fresh pork during storage. Meat Sci. (2016) 117:130–6. 10.1016/j.meatsci.2016.03.002 26971309

[3] Sowbhagya HB. Value-added processing of by-products from spice industry. Food Qual Saf. (2019) 3:73–80. 10.1093/fqsafe/fyy029

[4] Procopio FR Ferraz MC Paulino BN do Amaral Sobral PJ Hubinger MD. Spice oleoresins as value-added ingredient for food industry: Recent advances and perspectives. Trends Food Sci Technol. (2022) 122:123–139. 10.1016/j.tifs.2022.02.010

[5] Nagavekar N Singhal RS. Enhanced extraction of oleoresin from Piper nigrum by supercritical carbon dioxide using ethanol as a co-solvent and its bioactivity profile. J Food Process Eng. (2018) 41:e12670. 10.1111/jfpe.12670

[6] Luo H Li Z Straight CR Wang Q Zhou J Sun Y. Black pepper and vegetable oil-based emulsion synergistically enhance carotenoid bioavailability of raw vegetables in humans. Food Chem. (2021) 373:131277. 10.1016/j.foodchem.2021.131277 34799132

[7] Bui TT Fan Y Piao CH Van Nguyen T Shin DU Jung SY. (2020). *Piper Nigrum* extract improves OVA-induced nasal epithelial barrier dysfunction via activating Nrf2/HO-1 signaling. Cell Immunol. 351:104035. 10.1016/j.cellimm.2019.104035 32051090

[8] Tedasen A Khoka A Madla S Sriwiriyajan S Graidist P. Anticancer effects of piperine-free *Piper nigrum* extract on cholangiocarcinoma cell lines. Pharmacogn Mag. (2020) 16:28. 10.4103/pm.pm_288_19

[9] Zarai Z Boujelbene E Salem NB Gargouri Y Sayari A. Antioxidant and antimicrobial activities of various solvent extracts, piperine and piperic acid from *Piper nigrum*. LWT-Food Sci Technol. (2013) 50:634–641. 10.1016/j.lwt.2012.07.036

[10] Zinjarde SS Bhargava SY Kumar AR. Potent alpha-amylase inhibitory activity of Indian Ayurvedic medicinal plants. BMC Complem Altern Med. (2011) 11:5. 10.1186/1472-6882-11-5 21251279 PMC3037352

[11] Choudhary P Chakdar H Singh D Selvaraj C Saxena AK. Computational studies reveal piperine, the predominant oleoresin of black pepper (*Piper nigrum*) as a potential inhibitor of SARS-CoV-2 (COVID-19). Curr Sci. (2020) 119:1333–42. 10.18520/cs/v119/i8/1333-1342

[12] Olalere OA Abdurahman NH Alara OR Habeeb OA. Parametric optimization of microwave reflux extraction of spice oleoresin from white pepper (*Piper nigrum*). J Anal Sci Technol. (2017) 8:8. 10.1186/s40543-017-0118-9

[13] Haq IU Imran M Nadeem M Tufail T Gondal TA Mubarak MS. Piperine: A review of its biological effects. Phytother Res. (2021) 35:680–700. 10.1002/ptr.6855 32929825

[14] Moldovan ML Iurian S Puscas C Silaghi-Dumitrescu R Hanganu D Bogdan C. A design of experiments strategy to enhance the recovery of polyphenolic compounds from vitis vinifera by-products through heat reflux extraction. Biomolecules. (2019) 9:529. 10.3390/biom9100529 31557922 PMC6843815

[15] Lianfu Z Zelong L. Optimization and comparison of ultrasound/microwave assisted extraction (UMAE) and ultrasonic assisted extraction (UAE) of lycopene from tomatoes. Ultrason Sonochem. (2008) 15:731–7. 10.1016/j.ultsonch.2007.12.001 18226944

[16] Nurhadi B Saputra RA Setiawati TA Husein SN Faressi FR Utari CD. Comparison of *Curcuma domestica* and *Curcuma xanthorrhiza* oleoresins extracted using maceration, Soxhlet, and ultrasound-assisted extraction (UAE). Earth Environ Sci. (2020) 443:012074. 10.1088/1755-1315/443/1/012074

[17] Dutta S Bhattacharjee P. Nanoliposomal encapsulates of piperine-rich black pepper extract obtained by enzyme-assisted supercritical carbon dioxide extraction. J Food Eng. (2017) 201:49–56. 10.1016/j.jfoodeng.2017.01.006

[18] Lee J-G Chae Y Shin Y Kim Y-J. Chemical composition and antioxidant capacity of black pepper pericarp. Appl Biol Chem. (2020) 63:35. 10.1186/s13765-020-00521-1

[19] Aziz NS Sofian-Seng NS Wan Mustapha WA. Functional properties of oleoresin extracted from white pepper (*Piper nigrum* L.) retting waste water. Sains Malays. (2018) 47:2009–2015. 10.17576/jsm-2018-4709-08

[20] Chen Q Dong W Wei C Hu R Long Y. Combining integrated ultrasonic-microwave technique with ethanol to maximise extraction of green coffee oil from Arabica coffee beans. Ind Crops Prod. (2020) 151:112405. 10.1016/j.indcrop.2020.112405

[21] Hu W Huang C Wang M-H. Chemical composition, nutritional value, and antioxidant constituents of Kalopanax pictus leaves. Food Chem. (2012) 131:449–55. 10.1016/j.foodchem.2011.09.005

[22] Chanioti S Tzia C. Optimization of ultrasound-assisted extraction of oil from olive pomace using response surface technology: Oil recovery, unsaponifiable matter, total phenol content and antioxidant activity. LWT-Food Sci Technol. (2017) 79:178–89. 10.1016/j.lwt.2017.01.029

[23] Kusuma HS Mahfud M. Comparison of kinetic models of oil extraction from sandalwood by microwave-assisted hydrodistillation. Int Food Res J. (2017) 24:1697–702.

[24] Wizi J Wang L Hou X Tao Y Ma B Yang Y. Ultrasound-microwave assisted extraction of natural colorants from sorghum husk with different solvents. Ind Crops Prod. (2018) 120:203–13. 10.1016/j.indcrop.2018.04.068

[25] Jacotet-Navarro M Rombaut N Fabiano-Tixier AS Danguien M Bily A Chemat F. Ultrasound versus microwave as green processes for extraction of rosmarinic, carnosic and ursolic acids from rosemary. Ultrason Sonochem. (2015) 27:102–9. 10.1016/j.ultsonch.2015.05.006 26186826

[26] Gorgani L Mohammadi M Najafpour GD Nikzad M. Sequential microwave-ultrasound-assisted extraction for isolation of pperine from black pepper (*Piper nigrum* L.). Food Bioprocess Technol. (2017) 10:2199–2207. 10.1007/s11947-017-1994-0

[27] Sharifzadeh S Karimi S Abbasi H Assari M. Sequential ultrasound-microwave technique as an efficient method for extraction of essential oil from *Lavandula coronopifolia Poir*. J Food Meas Charact. (2022) 16:377–90. 10.1007/s11694-021-01170-8

[28] Xu S-Y Liu J-P Huang X Du L-P Shi F-L Dong R. Ultrasonic-microwave assisted extraction, characterization and biological activity of pectin from jackfruit peel. LWT-Food Sci Technol. (2018) 90:577–82. 10.1016/j.lwt.2018.01.007

[29] Garcia-Vaquero M Ummat V Tiwari B Rajauria G. Exploring ultrasound, microwave and ultrasound–microwave assisted extraction technologies to increase the extraction of bioactive compounds and antioxidants from brown macroalgae. Mar Drugs. (2020) 18. 10.3390/md18030172 32244865 PMC7142542

[30] Durović S Nikolić B Luković N Jovanović J Stefanović A Šekuljica N. The impact of high-power ultrasound and microwave on the phenolic acid profile and antioxidant activity of the extract from yellow soybean seeds. Ind Crops Prod. (2018) 122:223–231. 10.1016/j.indcrop.2018.05.078

[31] Rodinkov OV Smirnova EA Moskvin LN. Effect of temperature on the performance characteristics of continuous chromatomembrane gas extraction. J Anal Chem. (2015) 70:87–91. 10.1134/S106193481501013X

[32] Raman G Gaikar VG. Microwave-Assisted Extraction of Piperine from *Piper nigrum*. Ind Eng Chem Res. (2002) 41:2521–8. 10.1021/ie010359b

[33] Guo Z Zhao B Li H Miao S Zheng B. Optimization of ultrasound-microwave synergistic extraction of prebiotic oligosaccharides from sweet potatoes (*Ipomoea batatas* L.). Innovative Food Sci Emerging Technol. (2019) 54:51–63. 10.1016/j.ifset.2019.03.009

[34] Devi A Dwibedi V Khan ZA. Natural antioxidants in new age-related diseases. Rev Bras Farmacogn. (2021) 31:387–407. 10.1007/s43450-021-00175-0

[35] Zhang Q-A Shen H Fan X-H Shen Y Wang X Song Y. Changes of gallic acid mediated by ultrasound in a model extraction solution. Ultrason Sonochem. (2015) 22:149–54. 10.1016/j.ultsonch.2014.06.010 24974004

[36] Wang Y Li R Jiang Z-T Tan J Tang S-H Li T-T. Green and solvent-free simultaneous ultrasonic-microwave assisted extraction of essential oil from white and black peppers. Ind Crops Prod. (2018) 114:164–72. 10.1016/j.indcrop.2018.02.002

[37] Quaisie J Ma H Golly MK Tuly JA Amaglo NK Jiaqi Z. Effect of ultrasound-microwave irradiation hybrid technique on extraction, physicochemical, antioxidative, and structural properties of stearic acid-rich *Allanblackia parviflora* seed oil. Chem Pap. (2021) 75:4527–41. 10.1007/s11696-021-01666-z

[38] Yu J Lou Q Zheng X Cui Z Fu J. Sequential combination of microwave- and ultrasound-assisted extraction of total flavonoids from *Osmanthus fragrans* lour. Flowers Molec. (2017) 22:2216. 10.3390/molecules22122216 29236089 PMC6149695

[39] Carp OE Moraru A Pinteala M Arvinte A. Electrochemical behaviour of piperine. Comparison with control antioxidants. Food Chem. (2021) 339:128110. 10.1016/j.foodchem.2020.128110 33152887

[40] Quilaqueo M Millao S Luzardo-Ocampo I Campos-Vega R Acevedo F Shene C. Inclusion of piperine in β-cyclodextrin complexes improves their bioaccessibility and in vitro antioxidant capacity. Food Hydrocoll. (2019) 91:143–52. 10.1016/j.foodhyd.2019.01.011

[41] Yang Y Zhuang H Yoon S-C Wang W Jiang H Jia B. Rapid classification of intact chicken breast fillets by predicting principal component score of quality traits with visible/near-Infrared spectroscopy. Food Chem. (2018) 244:184–9. 10.1016/j.foodchem.2017.09.148 29120769

[42] Olalere OA Abdurahman NH Alara OR. Extraction, radical scavenging activities and physicochemical fingerprints of black pepper (*Piper nigrum*) extract. J Food Meas Charact. (2017) 11:2195–201. 10.1007/s11694-017-9604-4

[43] Vinatoru M Mason TJ Calinescu I. Ultrasonically assisted extraction (UAE) and microwave assisted extraction (MAE) of functional compounds from plant materials. TrAC, Trends Anal Chem. (2017) 97:159–78. 10.1016/j.trac.2017.09.002

[44] Shen S Zhou C Zeng Y Zhang H Hossen MA Dai J. Structures, physicochemical and bioactive properties of polysaccharides extracted from *Panax notoginseng* using ultrasonic/microwave-assisted extraction. LWT-Food Sci Technol. (2022) 154:112446. 10.1016/j.lwt.2021.112446

[45] Olalere OA Abdurahman HN Yunus RBM Alara OR Ahmad MM Zaki YH. Parameter study, antioxidant activities, morphological and functional characteristics in microwave extraction of medicinal oleoresins from black and white pepper. J Taibah Univ Sci. (2018) 12:730–7. 10.1080/16583655.2018.1515323

[46] Fernández-Ronco MP Gracia I De Lucas A Rodríguez JF. Extraction of *Capsicum annuum* oleoresin by maceration and ultrasound-assisted extraction: influence of parameters and process modeling. J Food Process Eng. (2013) 36:343–52. 10.1111/j.1745-4530.2012.00702.x

[47] Kusuma HS Mahfud M. RETRACTED: Preliminary study: Kinetics of oil extraction from basil (*Ocimum basilicum*) by microwave-assisted hydrodistillation and solvent-free microwave extraction. S Afr J Chem Eng. (2016) 21:49–53. 10.1016/j.sajce.2016.06.001

[48] Yedhu Krishnan R Rajan KS. Microwave assisted extraction of flavonoids from *Terminalia bellerica*: Study of kinetics and thermodynamics. Sep Purif Technol. (2016) 157:169–78. 10.1016/j.seppur.2015.11.035

[49] Kurniasari L Kusumo P. Kinetics of cinnamon oleoresin extraction using microwave-assisted extractor. J Phys. (2019) 1295:012014. 10.1088/1742-6596/1295/1/012014

